# Diagnostic Accuracy of High-Resolution Computed Tomography (HRCT) in Detecting Pneumocystis carinii in Renal Transplant Patients With Pulmonary Infection: A Study Using Bronchoalveolar Lavage As the Gold Standard

**DOI:** 10.7759/cureus.74831

**Published:** 2024-11-30

**Authors:** Kamran Fazal, Azeemuddin Muhammad, Muhammad A Sattar, Junaid Iqbal, Irfan Siddiqui, Adnan Fazal

**Affiliations:** 1 Radiology, Aga Khan University Hospital, Karachi, PAK; 2 Radiology, Sindh Institute of Urology and Transplantation, Karachi, PAK; 3 Emergency Department, Ziauddin University, Karachi, PAK; 4 Cardiology, National Institute of Cardiovascular Diseases, Karachi, PAK

**Keywords:** bronchoalveolar lavage, high-resolution ct scan, immune systems, pneumocystis carinii, renal transplant

## Abstract

Introduction: Patients receiving renal transplants have weakened immune systems and are more vulnerable to lung infections.

Objectives: To determine the diagnostic accuracy of high-resolution computed tomography (HRCT) in detecting pneumocystis carinii in renal transplant patients presenting with pulmonary infection in a tertiary care transplant center, keeping bronchoalveolar lavage (BAL) as the gold standard.

Methods: This cross-sectional study was conducted at the Department of Radiology, Sindh Institute of Urology and Transplant, Karachi, from February 14, 2023, to August 13, 2023. Using a non-probability consecutive sampling technique, we enrolled 81 post-renal transplant patients, aged 20 to 60 years, of both genders, who were receiving immunosuppressive therapy and referred to the Radiology Department for HRCT as part of a workup for pulmonary infection. Patients presented with pulmonary infection underwent HRCT and BAL. Diagnostic accuracy was determined and data was analyzed.

Results: In this study, 81 patients were enrolled with a mean age of 35.4±11.3 years. There were 56 (69.1%) male and 25 (30.9%) female patients. The mean duration of renal transplant was 18.09±12.8 months. The mean duration of symptoms was 5.32±2 days. Diabetes was present in 43 (53.1%) patients. The sensitivity of HRCT in diagnosing pneumocystis carinii was 93%, specificity was 91.7%, positive predictive value was 96.4%, negative predictive value was 84.6%, and diagnostic accuracy was 92.5%, taking BAL as the gold standard.

Conclusion: HRCT is a good imaging modality to diagnose pneumocystis carinii in renal transplant patients.

## Introduction

Every year, 18,000 individuals in Pakistan suffer from renal failure [1]. Since kidney transplantation considerably improves patient survival and quality of life over dialysis, it is regarded as the gold standard treatment for patients with end-stage renal illness [1]. Even with all of the advancements over the years, there are still many challenging clinical issues with patient management during post-transplant follow-up [2]. Acute rejection prevention is critical to the success of a kidney transplant, and modern immunosuppressive medication significantly improves transplant outcomes by lowering the frequency of acute rejection [2-4].

Both the donor and the recipient go through human leukocyte antigen (HLA) matching, complete blood counts, microbiological tests, and blood group matching in order to rule out any active infections. Comprehensive biochemical analyses are also carried out to rule out any organ dysfunction [5]. Immunosuppression is necessary for the receiver after matching in order to lower graft-versus-host disease (GVHD) [5,6]. Lifelong immunosuppression following transplantation puts the recipient at risk for infections. Infections account for three to four of the common pulmonary problems experienced by immunocompromised renal transplant recipients [7,8]. Given the high morbidity and mortality rates associated with lung infections, it is imperative to diagnose and treat them as soon as possible. While bronchoscopy and bronchoalveolar lavage (BAL) are invasive procedures used for microbiological confirmation, high-resolution computed tomography (HRCT) is a key diagnostic tool when used in the right clinical setting [9,10]. HRCT is the preferred imaging modality for immunosuppressed post-renal transplant patients with acute or subacute respiratory symptoms [11,12]. The goal of this research is to ascertain the diagnostic precision of HRCT in pneumocystis carinii pneumonia (PCP) diagnosis using BAL as the gold standard. Patients who underwent renal transplants participated in this study, which aided in PCP management for these patients. A more thorough and rapid recovery is the outcome of an accurate diagnosis and treatment. Data from this trial validated the diagnostic accuracy of HRCT in identifying pneumocystis carinii in recipients of renal transplants.

## Materials and methods

This cross-sectional study was conducted at the Department of Radiology, Sindh Institute of Urology and Transplant, Karachi, from February 14, 2023, to August 13, 2023. The sample size of 81 patients was calculated using a sample size calculator (by Wan Nor Arifin) for diagnostic accuracy, with a 95% confidence interval, a disease prevalence of 32.3%, a sensitivity assumed to be 95%, a 5% margin of error for sensitivity, specificity of 83%, and a 10% margin of error for specificity. Using a non-probability consecutive sampling method, we enrolled patients aged 20 to 60 years, of both genders, who were post-renal transplant recipients receiving immunosuppressive therapy for at least 4 weeks and referred to the Radiology Department for HRCT as part of the workup for pulmonary infection, as per the operational definition. We excluded individuals suffering from pulmonary injuries connected to transfusions based on the patient's medical history and record, patients suffering from obstructive respiratory disease, and individuals who have lung fibrosis as a result of immunosuppressive medication based on the patient's medical history.

After approval from the hospital's ethical board, patients fulfilling the inclusion criteria (underwent renal transplantation and presented with signs and symptoms of pulmonary infection) were enrolled from the Radiology Department of Sindh Institute of Urology and Transplant after being referred from the Urology Department for HRCT once pulmonary infection was diagnosed. A written informed consent was taken after explaining the purpose of the study. Demographic data regarding age, gender, educational level, socioeconomic status, diabetes (random blood sugar more than 200 mg/dL), hypertension (blood pressure more than 130/90mmHg), smoking, obesity (body mass index more than 25 kg/m^2^) and duration of 40 renal transplants was noted. A complete history was taken, and a physical examination was done. The patients were scanned by Siemens Healthcare SOMATOM computed tomography (CT) scanner. The chest CT images were obtained from the lung apices through the bases, and multiplanar reconstructions (MPR) were performed. HRCT images were analyzed for PCP according to the operational definition, where unenhanced HRCT showing a predominant ground-glass pattern in the upper fields, with or without associated reticulations and small cystic lesions, was labeled as positive for PCP.

Data was enrolled and analyzed by using SPSS version 22.0 (IBM Corp., Armonk, NY). Mean and standard deviation were calculated for quantitative variables like age, duration of renal transplant, and duration of disease. Frequency and percentage were calculated for categorical variables like gender, educational status, socioeconomic status, diabetes, hypertension, obesity, smoking, PCP diagnosed by HRCT, and BAL PCR. Diagnostic accuracy was determined in a 2x2 table. Effect modifiers like age, gender, duration of diseases, duration of renal transplant, educational status, socioeconomic status, diabetes, hypertension, obesity, and smoking were addressed through stratification of data for possible etiology of pulmonary infection. Post-stratification chi-square test was applied; in cases where the frequency was ≤5, Fisher's exact test was used. A p-value of ≤0.05 was considered statistically significant.

## Results

A total of 81 individuals with a mean age of 35.4±11.3 years were enrolled in our study, all of whom had undergone renal transplants, with an average duration of 18.0±12.8 months post-transplant. The symptoms persisted for 5.3±2 days on average as shown in Table 1.

**Table 1:** Descriptive statistics of demographic characteristics

Variable	Range	Mean
Age (years)	20-60	35.4±11.3
Duration of renal transplant (months)	4-46	18.0±12.8
Duration of symptoms (days)	3-10	5.3±2

Among them, 30.9% (25) were female and 69.1% (56) were male, while 53.1% (43) of the patients had diabetes and 61.7%(50) were hypertensive. Of the patients, 67.9% (55) were obese, and 53.1% (43) patients had a history of smoking, as shown in Table 2.

**Table 2:** Descriptive statistics of variables

Variable	Frequency	Percentage
Gender		
Male	56	69.1
Female	25	30.9
Diabetes		
Yes	43	53.1
No	38	46.9
Hypertension		
Yes	50	61.7
No	31	38.3
Obesity		
Yes	55	67.9
No	26	32.1
Smoking		
Yes	43	53.1
No	38	46.9

Using BAL as the gold standard, the sensitivity of HRCT in the diagnosis of PCP was 93%, specificity was 91.7%, positive predictive value was 96.4%, negative predictive value was 84.6%, and diagnostic accuracy was 92.5%, as shown in Table 3 and Table 4.

**Table 3:** Stratification of predictive variables with HRCT taking BAL as the gold standard HRCT, high-resolution computed tomography; BAL, bronchoalveolar lavage; PCP, pneumocystis carinii pneumonia

			PCP diagnosed by BAL		Total
			Yes	No	
PCP diagnosed by HRCT	Yes	Count	53	2	55
		PCP diagnosed on HRCT	96.4%	3.6%	100.0%
		PCP diagnosed on BAL	93.0%	8.3%	67.9%
	No	Count	4	22	26
		PCP diagnosed on HRCT	15.4%	84.6%	100.0%
		PCP diagnosed on BAL	7.0%	91.7%	32.1%

**Table 4:** Percentages of predictive variables for accuracy of HRCT HRCT, high-resolution computed tomography

Variables for accuracy of HRCT	Percentages
Sensitivity	93%
Specificity	91.7%
Positive predictive value	96.4%
Negative predictive value	84.6%
Diagnostic accuracy	92.5%

## Discussion

Numerous pulmonary problems are common in immunocompromised transplant recipients, with infections accounting for three-fourths of these cases. Given the high morbidity and mortality rates associated with lung infections, it is imperative to diagnose and treat them as soon as possible [12,13]. The length of time since a kidney transplant is one of the significant variables influencing the range of infections. Selecting the best course of action is greatly aided by early infection diagnosis and reduction of differential diagnosis possibilities [14,15]. Individuals with primary immunological deficits and those with kidney transplant diseases taking immuno-modulating medications (such as high-dose corticosteroids) are more susceptible to PCP. Due to the challenges in diagnosing PCP, a large number of cases have been diagnosed based only on clinical suspicion. Clinically, individuals have bilateral chest infections; they may or may not exhibit significant respiratory symptoms. On a chest X-ray, radiological symptoms are typically nonspecific; however, when paired with PCP risk factors, they are typically utilized to start therapy (trimethoprim/sulfamethoxazole, or TMP/SMX) while a formal diagnosis is being made [16,17].

A total of 81 individuals with a mean age of 35.4±11.3 years were enrolled in our study. Of the patients, 30.9%(25) were female and 69.1% (56) were male. The mean length of time for a kidney transplant was 18.0±12.8 months. The symptoms persisted for an average of 5.3±2 days. 53.1% (43) of the patients had diabetes. Sixty-one percent of the patients had hypertension (50). Of the patients, 67.9%(55) had obesity. 53.1%(43) of the patients were smokers. Thirty-two percent of patients have completed secondary education. The majority of patients (59.3%) were from the middle class. The HRCT demonstrated a sensitivity of 93%, specificity of 91.7%, positive predictive value of 96.4%, negative predictive value of 84.6%, and diagnostic accuracy of 92.5% in the diagnosis of PCP considering BAL to be the benchmark.

The most common cause of pneumonia in people with impaired immune systems is pneumocystis carinii, which frequently has nonspecific signs and symptoms. BAL, an invasive technique, is used as a diagnostic tool. HRCT is a useful substitute for PCP. HRCT was found to have 100%, 83.3%, 90.5%, and 100% of sensitivity, specificity, positive predictive value, and negative predictive value for the diagnosis of PCP using BAL as the gold standard in a prior investigation [18]. 33 patients (four women and 29 men) with a median age of 39 years (range: 26-49 years) were included in another investigation. There was a 12% PCP incidence. HRCT had 100% sensitivity, 89% specificity, and 90% accuracy (p<0.005) [19]. There were no false-negative interpretations and five false-positive ones. For the diagnosis of PCP, HRCT is a non-invasive modality and has shown to be quite sensitive with high diagnostic accuracy. The most common observations included cystic lesions, areas of ground-glass attenuation, usually in the perihilar region or higher lobes, and some degree of septal thickening or reticulation [20]. Additional HRCT results are detailed in this infection, such as lymphadenopathies, nodules, consolidation, pleural effusion, and/or bronchiectasis. 

This study does have some notable limitations like a small sample size, and the study was conducted over a short time and may not capture long-term effects or trends of the disease. Future research with a more extensive sample size and longer study duration could fill the existing gaps and validate the conclusions of this study. The limitation of being a single-center study could be mitigated through a multi-institutional approach.

## Conclusions

For kidney transplant recipients, timely identification and treatment of PCP are critical due to their compromised immune systems. HRCT has emerged as a highly sensitive and specific modality for identifying this condition. Its ability to provide detailed lung images aids significantly in the early detection and management of this potentially life-threatening infection.
